# Enhancement of Metformin’s Efficacy by Betel Nut Polyphenols in Type 2 Diabetic Rats Exposed to High Altitude

**DOI:** 10.2174/0118715303429512251203124106

**Published:** 2026-06-03

**Authors:** Xiaojing Zhang, Lin Luo, Wenbin Li, Hongfang Mu, Rong Wang

**Affiliations:** 1 Department of Clinical Pharmacy, The 940^th^ Hospital of Joint Logistic Support Force of Chinese People’s Liberation Army, Lanzhou, China;; 2 Department of Pharmacy, The 940^th^ Hospital of Joint Logistic Support Force of Chinese People’s Liberation Army, Lanzhou, China

**Keywords:** High-altitude environment, diabetes mellitus, metformin, betel nut polyphenols, oxidative stress, inflammatory factors

## Abstract

**Introduction:**

High-altitude environment inhibited the metabolism and increased the absorption of metformin in Type 2 Diabetes Mellitus (T2DM), but the therapeutic effect of metformin was impaired. This study aimed to investigate the effect of betel nut polyphenols (BP) on the therapeutic efficacy of metformin at high altitude.

**Methods:**

Firstly, we prepared the T2DM rat model by combining a high-sugar/high-fat diet with streptozotocin. For the pharmacokinetic (PK) study, 12 diabetic rats were randomly divided into the model + metformin (DMM) group and the DMM + BP (DMMB) group. Thirty-two diabetic rats were randomly divided into four groups for the pharmacodynamic (PD) study: model (DM) group, DM + metformin (DMM) group, DMM + high altitude (DMMH) group, and DMMH + BP (DMMHB) group, with the normal diet (DN) group as the control. Subsequently, fasting glucose, insulin resistance index (HOMA-IR), lactic acid (LA), pyruvate (PA), lactate dehydrogenase (LDH), oxidative stress, and inflammatory factors were measured in all groups.

**Results:**

The PK results showed that BP did not affect the PK behaviour of metformin *in vivo* in T2DM rats. The PD results showed that, compared with the DMM group, fasting glucose and HOMA-IR were significantly higher in the DMMH group. Meanwhile, PA, LA, and LDH levels were significantly increased, the metformin content in liver tissue was significantly decreased, and oxidative stress and inflammatory damage were aggravated at high altitude. After treatment with BP, the beneficial effects of metformin were enhanced, the content of metformin in liver tissue was significantly increased, and oxidative stress and inflammatory damage were ameliorated in the DMMHB group.

**Discussion:**

The therapeutic effect of metformin was impaired in a high-altitude environment. This study confirmed that the beneficial effects of metformin were enhanced when it was co-administered with the anti-hypoxic drug BP in a high-altitude environment, providing a new therapeutic approach for treating diabetic patients in such environments.

**Conclusion:**

BP obviously enhanced the therapeutic effect of metformin by improving the antioxidant capacity of T2DM rats in a high-altitude environment. Therefore, BP can serve as a potential adjuvant to enhance the beneficial effects of metformin in a high-altitude environment.

## 1 INTRODUCTION

High altitude can be divided into high altitude (2000 to 4000 m), very high altitude (4000 to 5500 m), and extremely high altitude (>5500 m) [1, 2]. More than 140 million individuals around the world live at high altitudes [2], and more than 100 million lowlanders visit mountainous areas above 2500 m annually [3]. A high-altitude environment is characterized by strong radiation, cold, low pressure, hypoxia, and so on [4]. Hypoxia is the most important factor affecting the physiology and normal activities of the body [5]. Hypoxia causes various negative impacts on organs, including physiological, hematological, and molecular changes to adapt to extreme environmental conditions [2]. High altitude can lead to oxidative stress [6, 7]. Oxidative stress is a damaging process that can severely alter cell membranes and cellular contents, such as lipids, lipoproteins, proteins, and DNA [8]. Numerous studies have demonstrated a strong correlation between oxidative stress and high-altitude diseases such as acute mountain sickness [9, 10], high-altitude pulmonary edema [9, 11, 12], and high-altitude cerebral edema [9, 13]. At the same time, short- or long-term exposure can induce respiratory [14, 15], cardiovascular [5, 16], and endocrine dysfunction [17] at high altitude.

High-altitude trekking can expose people to extreme environmental conditions, which makes it more difficult for people with diabetes to control their glycaemia [18]. Diabetes mellitus is a chronic metabolic disease characterized by persistent hyperglycemia [19-21]. The two major forms are type 1 diabetes mellitus, caused by autoimmune destruction of pancreatic β-cells, and type 2 Diabetes Mellitus (T2DM), which results from a combination of insulin resistance and progressive β-cell dysfunction [22, 23]. As β-cell failure plays a key role in T2DM progression, preserving β-cell function is an emerging therapeutic focus [24, 25]. Liver-targeted FGF7 particles have been shown to enhance β-cell engraftment and improve glucose control in type 1 diabetes models [26], underscoring the potential of β-cell-focused interventions. Currently, metformin remains the first-line oral hypoglycemic agent in the clinical management of T2DM, acting primarily by reducing hepatic glucose production and improving insulin sensitivity [27, 28]. However, metformin may increase blood lactic acid (LA) levels and thus increase the risk of lactic acidosis in patients with diabetes mellitus who are taking it under certain conditions, such as hypoxia, renal, and cardiac insufficiency [29]. Zhu *et al*. [30] showed that metformin metabolism was slowed and its absorption increased in normal rats at simulated high altitude. Shen *et al.* [31] also showed that metformin metabolism was slowed and absorption increased in T2DM patients, but the therapeutic effect of metformin was reduced at high altitude. Therefore, it is crucial to find ways to enhance the positive effects of metformin in a high-altitude environment.

Betel nut is widely distributed and has been used in traditional Chinese medicine for more than 10,000 years; it is also one of the four most important medicinal herbs in southern China [32]. The main constituents of betel nut are polyphenols, fibres, polysaccharides, fatty acids, and alkaloids [32, 33], which have a variety of biologically active functions, such as antioxidant, anti-inflammatory, antiviral, and anti-tumor [32, 34], indicating that betel nut has good medicinal value. Betel nuts contain multiple chemical constituents, with over 60 compounds isolated from them, including polyphenols (31.1%), polysaccharides (18.7%), fatty acids (14.0%), and other substances [35]. Polyphenols are the most common antioxidant phytochemicals [36] and have been shown to have many pharmacological activities, including anti-fatigue, antioxidant, anti-inflammatory, and analgesic effects [37]. Consequently, they possess beneficial physiological effects. Based on their chemical structures, polyphenols can be categorized into flavonoids and non-flavonoids. The primary chemical constituents of flavonoids are catechin, epicatechin, gallocatechin gallate, isorhamnetin, and luteolin. The primary chemical constituents of non-flavonoids are vanillic acid and ferulic acid [35]. BP constitute a significant proportion of betel nut and hold research value for their antioxidant and anti-hypoxic properties. Yi *et al.* [38] demonstrated that BP reduced lipopolysaccharide-stimulated oxidative stress *via* the MAPK-mediated Nrf2/HO-1 signaling pathway. BP regulated redox balance by up-regulating the expression of oxidoreductase-related proteins [39].

BP has strong anti-hypoxia activity [38-40]. Previous research by our group showed that BP significantly improves blood gas status in high-altitude rats, reduces inflammation and oxidative stress, and protects various organs under hypoxic conditions [41], indicating its potential as an antioxidant agent in a high-altitude environment. Based on this, we established a T2DM rat model using a high-sugar/high-fat diet combined with streptozotocin and investigated whether BP could enhance the therapeutic effect of metformin under high-altitude conditions. This study aimed to evaluate the impact of BP on fasting glucose, HOMA-IR, oxidative stress, and inflammatory markers in T2DM rats exposed to hypoxia.

## 2 MATERIALS AND METHODS

### 2.1 Chemicals and Reagents

Metformin hydrochloride (purity ≥ 98%, Cat: B25331) and ranitidine hydrochloride (purity ≥ 98%, Cat: A10007) were purchased from Shanghai Yuanye Bio-Technology Co., Ltd. (Shanghai, China). The internal standard (IS) used was ranitidine hydrochloride. Metformin hydrochloride tablets (specification: 0.5 g/tablet × 20 tablets, approval number: H20023370) were purchased from Sino-American Shanghai Squibb Pharmaceutical Ltd. (Shanghai, China). Malondialdehyde (MDA) assay kit (Cat: A003-1-2), reduced glutathione (GSH) assay kit (Cat: A006-2-1), superoxide dismutase (SOD) assay kit (Cat: A001-3-2), pyruvate (PA) assay kit (Cat: A081-1-1), LA assay kit (Cat: A019-2-1), lactate dehydrogenase (LDH ) assay kit (Cat: A020-2-2), and insulin assay kit (Cat: H203-1-2) were purchased from Nanjing Jiancheng Bioengineering Institute Co., Ltd. (Nanjing, China). Rat interleukin-6 (IL-6) ELISA kit (Cat: MB-1731A) and rat monocyte chemoattractant protein-1 (MCP-1) ELISA kit (Cat: MB-6773A) were purchased from Jiangsu Meibiao Biotechnology Co., Ltd. (Jiangsu, China). Rat interleukin-1β (IL-1β) ELISA kit (Cat: JYM0419Ra) and rat tumor necrosis factor-α (TNF-α) ELISA kit (Cat: JYM0635Ra) were provided by Wuhan Jiyinmei Biotech Co., Ltd. (Wuhan, China).

### 2.2 Experimental Instruments and Conditions

API 3200 System and UFLC-20A high-performance liquid system were provided by AB SCIEX (Redwood City, CA, USA) and Shimadzu (Japan), respectively. The Gemini C18 column (75 mm×3 mm, 3 μm) was provided by Agela & Phenomenex (Tianjin, China). The data were collected in multiple reaction monitoring mode on the API 3200 system, using electrospray ionization in positive mode. The voltage of the ion spray was set to 4100 V. The ion source temperature was set to 350°C. The Declustering Potential (DP) and the Collision Energies (CE) of metformin were 22 V and 28 eV, respectively. The DP and the CE of IS were 15 V and 21 eV, respectively. The detection ion pair of metformin and IS was *m/z* 130.2→71.0 and *m/z* 315.2→176.0, respectively. The samples were eluted in isocratic mode. The elution was performed using a formic acid/acetonitrile/formic acid water solution (85:15, *V/V*) at a flow rate of 0.4 mL/min. The temperature of the column was set at 40°C.

### 2.3 Rat Plasma Samples Preparation

Metformin and IS standard stock solutions were prepared in methanol. The concentration of these solutions was 2 mg/mL and 0.2 mg/mL, respectively. They were stored at -20°C prior to use. The IS solution was diluted with the mobile phase to a final concentration of 0.5 µg/mL. This solution was then used as the IS working solution.

Protein precipitation was used to treat blank rat plasma or rat plasma containing metformin and IS. First, 5 µL of metformin solution was added to 45 µL of blank plasma, and the mixture was vortexed for 30 s. Then, 200 µL of the IS working solution was added to the plasma sample and vortexed for 60 s. The mixture was centrifuged at 12,000 × g for 15 min, after which a 2 µL sample of the supernatant was injected into the LC-MS/MS for analysis.

### 2.4 Experimental Design

#### 2.4.1 Preparation of BP

BP was provided by our research group [42]. The preparation method was as follows: 20 kg of betel nuts from the Traditional Chinese Medicine Pharmacy of the 940th Hospital of the Joint Logistic Support Force of China were crushed and passed through sieve No. 3. Then, 10, 8, and 6 volumes of 80% ethanol were added sequentially, followed by heat reflux extraction at 80°C three times for 2, 1.5, and 1.5 hours, respectively. The extracts were combined, filtered, and the ethanol was recovered under reduced pressure to obtain the crude extract.

The crude extract was suspended in an equal volume of water and loaded onto a macroporous adsorption resin column. Elution with 80% ethanol was performed, and the ethanol was recovered under reduced pressure, followed by vacuum drying at 60°C. The resulting extract was then suspended in water and applied to a polyamide chromatography column. Impurities were removed by elution with pure water, and the target components were eluted with 80% ethanol. The ethanol was removed under reduced pressure, and the extract was dried under vacuum at 60°C to obtain the final dried BP extract.

The total polyphenol content of BP was determined using the ferrous tartrate method, revealing a content of 52.7%.

#### 2.4.2 Animals

Male Wistar rats (180–220 g), aged six to eight weeks, were provided by SPF (Beijing) Biotechnology Co., Ltd. (Beijing, China). The animal room was maintained at 25 ± 2°C with an average humidity of 55 ± 5%. All animals were kept under standard conditions (12 h light/dark cycle) and were acclimatized for one week prior to the experiments. This study was approved by the Ethics Committee of the 940th Hospital of the Joint Logistic Support Force of the Chinese People’s Liberation Army (Certificate of Approval: 2021KYLL189).

#### 2.4.3 Establishment of T2DM Model

The T2DM model was established by combining a high- sugar/high-fat diet with streptozotocin, as described in previous studies [43-46]. In brief, eighty rats were fed a high-sugar/high-fat diet containing 24.2% protein, 42.1% carbohydrate, 25.4% fat, and 8.3% other ingredients (DM group), accounting for potential animal losses during the modeling process and the expected success rate of the model. After 28 days, these rats were injected with 30 mg/kg streptozotocin. Meanwhile, twenty-five rats were fed a normal diet (DN group) and injected with an equal volume of vehicle citrate buffer solution (pH 4.5) after the same feeding period.

After 3 days, they were fasted for 12 hours, and the fasting glucose and insulin were measured. When fasting glucose was ≥11.1 mmol/L, with polydipsia, polyphagia, polyuria, and insulin resistance, it indicated that the T2DM model had been successfully established and could be used for follow-up studies (the model's success rate was 85%). Second, model rats were randomly selected, and pancreatic tissue was extracted and stained with HE to observe pancreatic tissue damage. Finally, an oral glucose tolerance test was performed following an overnight fast. In brief, DN and DM rats were given 2 g/kg of 50% glucose solution by oral gavage after 12 hours of fasting, and blood glucose was measured before and at 30, 60, and 90 minutes after administration.

#### 2.4.4 Hypoglycemic Effect of BP

Twelve diabetic rats were randomly divided into a diabetes model (DM) group and a DM + BP (DMB) group (n = 6 per group). Rats in the DMB group received 800 mg/kg BP intragastrically once daily for seven consecutive days. Fasting glucose and body weight were measured before administration and on days 1, 3, 5, and 7 after administration. Meanwhile, the DN and DM groups received sterile water.

#### 2.4.5 Effect of BP on the Pharmacokinetic (PK) of Metformin

Twelve diabetic rats were randomly divided into a model + metformin (DMM) group and a DMM + BP (DMMB) group (n = 6 per group). The DMMB group received 800 mg/kg BP by intragastric gavage once daily for seven days, while the DMM group received sterile water by intragastric gavage. On the evening of day 7, the rats were fasted. The following morning, the DMM group was administered 80 mg/kg metformin hydrochloride, and the DMMB group was administered 80 mg/kg metformin hydrochloride along with 800 mg/kg BP.

Blood samples were collected from the orbital vein before administration and at 5, 15, 30, 45 minutes, and 1, 1.5, 2, 4, 6, 8, 12, and 24 hours after administration. The blood samples were centrifuged at 4°C and 3,000 × g for 10 minutes to collect plasma. Fifty microliters of plasma were transferred to a new centrifuge tube and subjected to a protein precipitation assay. A non-compartmental model was used to calculate the pharmacokinetic parameters of metformin in the DMM and DMMB groups using WinNonlin 8.1.

#### 2.4.6 Effect of BP on Pharmacodynamic (PD) of Metformin

##### 2.4.6.1 Groups and Processing Scheme

Thirty-two diabetic rats were randomly divided into four groups: model (DM) group, DM + metformin (DMM) group, DMM + high altitude (DMMH) group, DMMH + BP (DMMHB) group, with the normal diet (DN) group as the control (n = 8 per group). The DN, DM, and DMM groups were housed and experiments conducted at the Animal Laboratory of the 940th Hospital of the Joint Logistic Support Force of the Chinese People’s Liberation Army in Lanzhou, Gansu Province (altitude 1,500 m, oxygen content 18.55%). The DN and DM groups received sterile water, while the DMM group received 80 mg/kg metformin *via* gastric gavage for 7 days. The DMMHB group received 800 mg/kg BP for three days in Lanzhou.

On the morning of the fourth day, the DMMH and DMMHB groups were rapidly transported to the high-altitude laboratory in Yushu Autonomous Prefecture, Qinghai Province (altitude 4,010 m, oxygen content 12.70%), using temperature-controlled freight vehicles and air transport. The entire transport process was completed within 24 hours. Upon arrival in Yushu, the DMMH group was administered 80 mg/kg metformin for 7 days, while the DMMHB group received a combined dose of 80 mg/kg metformin and 800 mg/kg BP for 7 days.

##### 2.4.6.2 PD Studies

Body weight, fasting glucose, and blood samples were measured before administration and 7 days after, respectively. The blood samples were left to stand at room temperature for 0.5 hour, after which they were centrifuged at 3,000 ×g for 10 min to obtain serum for insulin measurement. After the last blood collection, the rats were sacrificed, and their liver tissues were taken.

Rat liver tissues were homogenised using a pre-cooled saline solution with a final concentration of 0.5 g/mL. A mixture of 50 µL of liver tissue homogenate and 300 µL of IS working solution was vortexed for 1 minute. The mixture was then centrifuged at 12,000 × g for 15 minutes, after which the clear layer was injected into the LC-MS/MS for analysis.

##### 2.4.6.3 Biochemical Analysis in Blood and Liver Tissue

The levels of PA, LA, and LDH in serum samples were determined strictly according to the assay kit instructions. Each sample was measured in triplicate.

Rat liver tissues were weighed and homogenized using pre-cooled saline (1/9, *W/V*). The mixture was centrifuged at 1,000 × g for 10 minutes, and the supernatant was used as the 10% homogenate supernatant for the assay. The levels of MDA, GSH, SOD, IL-1β, IL-6, TNF-α, and MCP-1 in liver tissue were strictly determined in accordance with the requirements of the MDA, GSH, SOD, IL-1β, IL-6, TNF-α, and MCP-1 assay kit. Each sample was measured in triplicate.

### 2.5 Statistical Analysis

All experimental results are expressed as mean ± standard deviation (mean ± SD). Statistical analyses were performed using GraphPad Prism version 10.1.2 (GraphPad Software, CA, USA). A two-tailed Student's t-test was used to analyze model establishment. Two-way ANOVA was applied to analyze the oral glucose tolerance test, the effect of BP on fasting glucose, and the pharmacodynamic study. One-way ANOVA was used to analyze the biochemical assays, followed by post-hoc testing. A *p*-value < 0.05 was considered statistically significant.

## 3 RESULTS

### 3.1 T2DM Model Establishment

DN and DM rats were fed a standard diet and a high-sugar/high-fat diet, respectively. There was no significant difference in fasting glucose between the two groups before streptozotocin injection; however, fasting glucose in T2DM rats increased significantly after streptozotocin injection (Fig. **1A**). Compared with the DN group, insulin secretion was significantly decreased, and the insulin resistance index (HOMA-IR, Equation (1) [47, 48]) was significantly increased in the DM group after streptozotocin injection (Fig. **1B** and **C**).

HE staining results showed that the boundary structure of pancreatic islet cells in DN rats was clear, whereas the boundary structure in DM rats was irregular, with a marked increase in blue collagen connective tissue deposition in the islets (Fig. **1E**). Additionally, the oral glucose tolerance test revealed that blood glucose levels in DN rats did not increase significantly after administration of 50% glucose solution, whereas blood glucose in DM rats increased significantly by 60.5%, 71.8%, and 48.1% at 30, 60, and 120 minutes, respectively (Fig. **1D**).

HOMA-IR= (fasting insulin× fasting glucose)/ 22.5 **(1)**

### 3.2 Effect of BP on Fasting Glucose

The DN group rats showed gradual weight gain without significant changes in fasting glucose. In contrast, the DM group exhibited a significant decrease in body weight and elevated blood glucose levels. After administration of BP to the DMB group, there were no significant differences in body weight or fasting glucose compared to before administration (Figs. **2A** and **B**). These results indicated that BP did not reduce fasting glucose levels in T2DM rats.

### 3.3 *In vivo* PK Studies Results

Typical chromatograms of metformin and ranitidine in rat plasma are shown in Fig. (**3A**). Both metformin and ranitidine were detected at 0.76 min, and the peaks used for analysis were not interfered with by other plasma components. A non-compartmental model was used to calculate the pharmacokinetic (PK) parameters of metformin in the DMM and DMMB groups. The mean plasma concentration–time curves and PK parameter results are shown in Fig. (**3B**) and Table **1**, respectively. The results indicate no significant differences in metformin PK parameters between the two groups. Therefore, BP had no effect on the *in vivo* PK of metformin in T2DM rats.

### 3.4 *In vivo* PD Studies Results

We primarily examined the pharmacodynamics (PD) of metformin in T2DM rats under high-altitude conditions following combined administration of metformin and BP. Body weight, fasting blood glucose, and HOMA-IR were used to evaluate PD; results are shown in Figs. (**4A**-**C**). In the DM group, body weight decreased significantly, while fasting glucose and HOMA-IR increased significantly. After metformin treatment, the DMM group showed a significant increase in body weight, along with decreases in fasting glucose and HOMA-IR.

In the high-altitude environment, administration of the same metformin dose resulted in reduced body weight and increased fasting glucose and HOMA-IR in the DMMH group. However, when BP was co-administered, the DMMHB group exhibited a significant increase in body weight and significant decreases in fasting glucose and HOMA-IR compared with the DMMH group. We also measured metformin concentration in liver tissue. The results showed that metformin levels were significantly decreased in the DMMH group, whereas co-administration with BP significantly increased hepatic metformin concentration in the DMMHB group (Fig. **5**).

### 3.5 Levels of LA, PA, and LDH in Serum

The levels of LA, PA, and LDH in rat serum were measured, with results shown in Figs. (**6A**-**C**). Diabetes induction in the DM group significantly increased LA, PA, and LDH levels compared with the DN group, whereas metformin treatment significantly reduced these levels in the DMM group. However, in the high-altitude environment, the same metformin dose in the DMMH group resulted in significantly higher levels of LA, PA, and LDH. Co-administration of BP significantly decreased LA, PA, and LDH levels in the DMMHB group.

### 3.6 Levels of Oxidative Stress in Liver Tissue

The results of oxidative stress in liver tissue are shown in Fig. (**7**). Diabetes induction in the DM group increased MDA content and decreased GSH content and SOD activity compared to the DN group. Compared with the DM group, metformin significantly reduced MDA content in the DMM group under normal oxygen conditions, whereas high-altitude conditions significantly increased MDA content in the DMMH group. After combining BP and metformin, we found that BP significantly reduced MDA levels (Fig. **7A**). Metformin significantly increased GSH content and SOD activity in the DMM group. SOD activity and GSH content in the DMMH group were obviously decreased at high altitude, but BP significantly increased the SOD activity and GSH content in the DMMHB group (Figs. **7B** and **7C**). These results indicated that BP can ameliorate the oxidative stress damage caused by a high-altitude environment.

### 3.7 Levels of Inflammatory Factors in Liver Tissue

The results of inflammatory factors in liver tissue were shown in Figs. (**8A-D**). Compared with the DN group, diabetes induction increased the levels of IL-6 and MCP-1 in the DM group. Metformin significantly reduced the levels of the inflammatory factors IL-6 and MCP-1 in the DMM group compared with the DM group. However, high altitude significantly increased inflammatory levels in the DMMH group, and BP significantly decreased the levels of IL-6, IL-1β, TNF-α, and MCP-1 in the DMMHB group. These results proved that BP can ameliorate the inflammatory damage caused by a high-altitude environment.

## 4 DISCUSSION

### 4.1 Model Validation and Rationale

Compared with the normal physiological state, pathological animal models serve as a basis for studying the PK and PD behaviour of drugs *in vivo* and provide a positive reference for clinical medication. In this study, we successfully created a T2DM model using a high-sugar/high-fat diet in combination with streptozotocin. Streptozotocin has been widely used to induce diabetes models [49]. The characteristics of streptozotocin are similar to those of human diabetes mellitus, including selective destruction of β-cells, insulin deficiency, hyperglycaemia, polydipsia, and polyuria [49]. A high-fat diet can initially cause rats to develop insulin resistance, reduce the dose of streptozotocin, reduce peripheral tissue toxicity, and yield a higher and more stable model formation rate [50]. After streptozotocin injection, fasting glucose increased sharply, insulin secretion decreased, and HOMA-IR increased in rats (Figs. **1A-C**). Meanwhile, the oral glucose tolerance test showed that the blood glucose of rats injected with streptozotocin was above 11.1 mmol/L at 2 hours after glucose administration. HE staining revealed pathological changes in pancreatic tissue. These results indicated that we had successfully established T2DM model.

### 4.2 Mechanistic Implications

Betel nut has many pharmacological activities, such as hypoglycemic, anti-inflammatory, and antioxidant effects [32, 37]. Previous research has shown that polyphenols can alleviate complications of diabetes, including cardiovascular disease and diabetic nephropathy [51]. The polyphenols in betel nut have been shown to lower blood pressure and possibly lower blood glucose, thereby helping to prevent complications [52]. Sun *et al.* [32] suggested that BP may be a good option for treating diabetes mellitus. This study first investigated the effects of BP on glucose in diabetic rats and found that BP did not exhibit hypoglycemic activity. A rapid and sensitive LC-MS/MS method was developed to analyse metformin quantitatively in rat plasma (Supplementary Tables **S1-S3**), and successfully applied this method to analyse the PK behaviour of metformin in two groups of rats. The PK parameters were calculated using a non-compartmental model, and the results showed that BP did not affect the PK behaviour of metformin *in vivo* in T2DM rats. Therefore, we ruled out the possibility that BP interferes with the beneficial effects of metformin by altering its PK and PD in T2DM rats.

### 4.3 Comparison with Prior Work

T2DM rats gained weight and reduced fasting glucose and HOMA-IR after administration of metformin. However, high altitude reversed the therapeutic effect of metformin in T2DM Rats, resulting in weight loss and increased fasting glucose and HOMA-IR. When metformin and BP were co-administered, BP significantly reversed the inhibitory effect of metformin in high altitude (Fig. **4**). T2DM accounts for over 90% of diabetes mellitus [53-55], and increased hepatic glucose production is the main cause of fasting hyperglycemia [56]. Metformin ameliorates hyperglycemia mainly by inhibiting hepatic gluconeogenesis [27, 57, 58]. We also examined metformin concentration in liver tissue and found that it was significantly reduced at high altitude, whereas BP significantly increased metformin accumulation. Notably, we observed that high-altitude exposure reduced metformin concentration in liver tissue, suggesting altered hepatic disposition under hypoxic stress. When metformin and BP were co-administered, BP enhanced metformin's therapeutic effect by increasing its concentration in liver tissue at high altitude. This aligns with hypotheses that improving hepatic insulin and glucose clearance can markedly influence systemic glycemia. A concept also invoked in bariatric surgery research, wherein enhanced hepatic clearance following surgery has been proposed to underlie early diabetes remission [59-62].

LA is an intermediate metabolite of glycolysis and is produced from PA under hypoxic conditions by LDH [29]. Diabetes mellitus induction can lead to tissue hypoxia and increased LA [63]. Metformin significantly reduced LA, PA, and LDH levels in the DMM group, indicating that it has a beneficial effect in the treatment of diabetes mellitus (Figs. **6A**-**C**). It has also been reported that the incidence of lactic acidosis is higher among diabetes mellitus patients taking metformin in high-altitude areas than in flat areas (17.65% *vs.* 2.50%), indicating that hypoxia exacerbates the adverse effects of metformin use [29]. Shen *et al*. [31] also studied serum LA levels in T2DM patients at sea level and at high altitude and found that serum LA levels at high altitude were elevated by 19.6%. The therapeutic effects of metformin also appear to be impaired at high altitude [29, 31]. This is consistent with our study, which showed that LA, PA, and LDH levels were significantly higher in the DMMH group after administration of the same metformin dose as in the DMM group. After BP administration, BP significantly reduced LA, PA, and LDH levels in the DMMHB group. Therefore, high altitude inhibited the effect of metformin and could increase the risk of patients developing lactic acidosis, but BP could reduce the incidence of this side effect.

Oxidative stress [64, 65] and chronic low-grade inflammation [8, 64, 66] underlie the pathogenesis of diabetes mellitus and the development of its complications. Oxidative stress, defined as an imbalance between prooxidants and antioxidants, is associated with many pathophysiological processes. Oxidative stress can exacerbate chronic diseases [67]. In diabetes mellitus, oxidative stress leads to reduced insulin production and impaired insulin secretion [68]. Oxidative stress reduces β-cell function and survival through oxidative damage and macromolecular modifications, disruption of antioxidant defense systems, and decreased expression of β-cell maturation genes [69]. Metformin exerts much of its glucose-lowering effect *via* hepatic mechanisms. When oxygen supply is reduced due to hypoxia at high altitude, the liver is more susceptible to oxidative stress [70]. GSH is often referred to as the “mother of all antioxidants” and is an important antioxidant [8]. SOD is the main antioxidant defence system. Lipid peroxides, such as MDA, can indirectly reflect the extent of cellular damage. Our results showed that compared with the DM group, metformin significantly reduced the MDA content, increased the SOD activity and GSH content in the DMM group, whereas metformin amelioration of oxidative stress injury in the DMMH group was largely suppressed in high altitude (Figs. **7A**-**C**), which is consistent with the results of the study by Zhao *et al.* [29]. After administration of BP, the MDA content was significantly reduced, while SOD activity and GSH content were significantly increased in the DMMHB group. Thus, BP could enhance the body's antioxidant capacity and alleviate oxidative stress damage. In addition to oxidative stress, inflammation plays a key role in the development of T2DM and its complications [71]. Most studies agree that the inflammatory response promotes the development of T2DM through insulin resistance [8]. Inflammation significantly increases insulin resistance and blood glucose levels, thereby directly affecting insulin resistance and blood glucose [8]. Oxidative stress injury induced by high altitude hypoxia. Oxidative stress has been linked to a variety of disease states, including inflammation [72] and liver injury [73]. Metformin reduced oxidative stress and/or inflammatory markers [68, 74], which is consistent with our findings. However, the levels of the inflammatory markers IL-6, IL-1β, MCP-1, and TNF-α in the liver tissue of the DMMH group were significantly increased. This suggested that high altitude impairs the beneficial effects of metformin on the body and that BP ameliorates the inflammatory damage caused by the high-altitude environment. Therefore, BP ameliorated inflammatory injury by increasing antioxidant capacity, thereby reducing oxidative stress at high altitude.

### 4.4 Limitations and Future Directions

Areca seeds are an excellent food material with potential antioxidant properties [75]. BP can ameliorate hypoxic injury in pulmonary microvascular endothelial cells by reducing oxidative stress and decreasing the expression of inflammation-related proteins [76]. Additionally, BP can prevent high-altitude exercise-induced fatigue by maintaining the normal physiological structure of lung tissue and enhancing resistance to oxidative stress [77]. The results of this study further demonstrated that BP significantly reduced oxidative stress and inflammatory injury induced by the high-altitude environment. Therefore, BP has great potential for development as a novel anti-high-altitude hypoxia agent with significant clinical value.

Nevertheless, several mechanistic uncertainties remain, such as how hypoxia alters hepatic transporter expression or affects hepatic microcirculation, and whether BP modulates these processes directly or indirectly. Future studies should investigate these mechanisms in greater detail. Functional imaging approaches, such as manganese-enhanced MRI, may provide a valuable, non-invasive method for assessing β-cell function and insulin secretion dynamics *in vivo* under hypoxic conditions. These techniques would allow longitudinal monitoring of pancreatic activity without the need for tissue collection or animal sacrifice, thereby enhancing the translational relevance of the findings.

## CONCLUSION

The high-altitude environment inhibited the beneficial effects of metformin by inducing oxidative stress and increasing inflammation, while BP significantly reduced the oxidative stress and inflammation induced by the high-altitude environment and thereby enhanced the therapeutic effects of metformin. Therefore, we believed that combining the antioxidant stress drug BP with the hypoglycemic drug metformin may be a new way to treat high-altitude diabetes mellitus. However, the effects of hypoxia on hepatic blood flow, transporter function, and microcirculation, and the manner in which BP modulates these processes, require in-depth investigation in future studies. Meanwhile, the long-term effects of BP and its potential side effects in humans need to be investigated in order to develop BP as an adjuvant drug.

## Figures and Tables

**Fig. (1):**
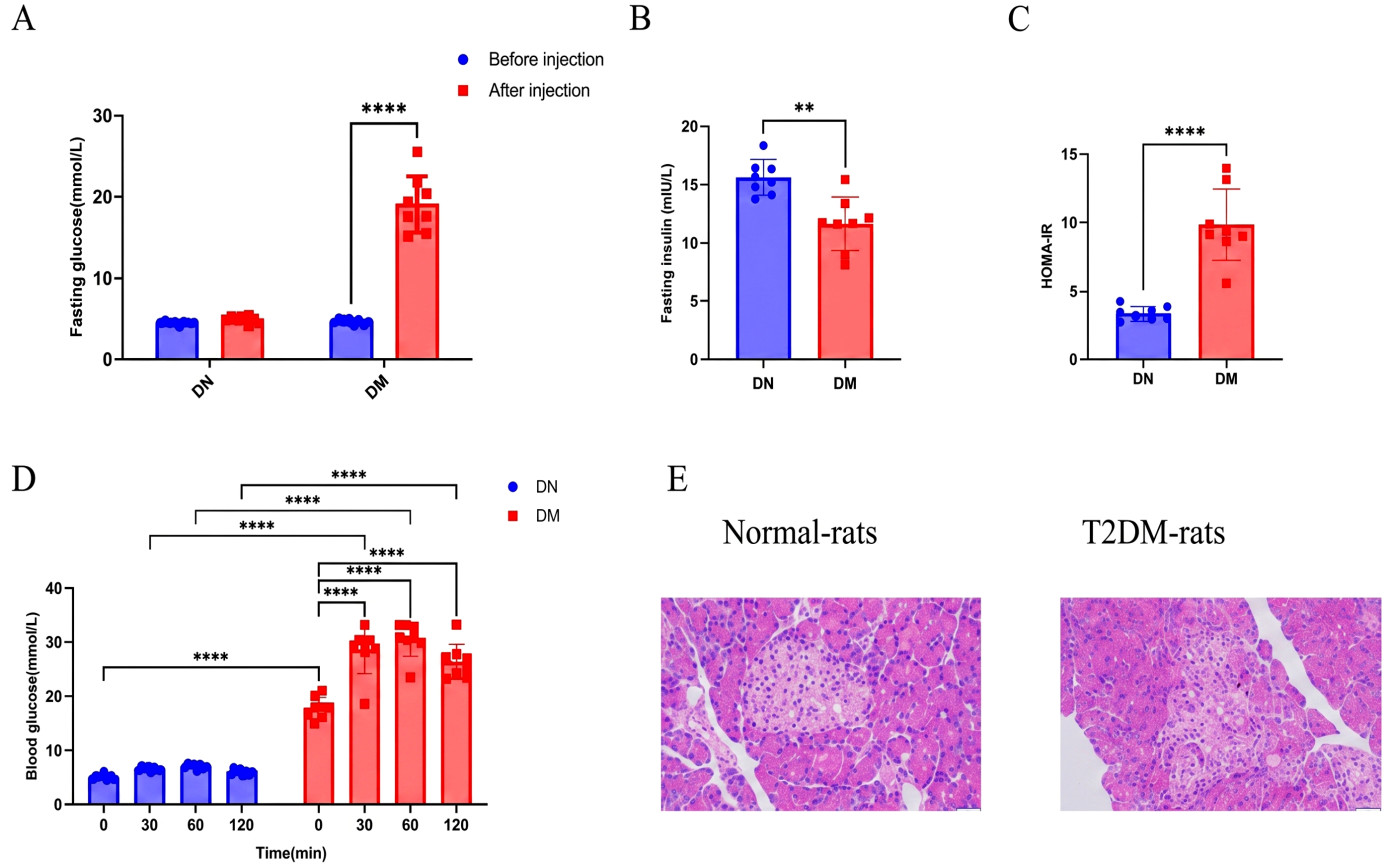
Different assessment indices of T2DM rats. **(A)** Fasting glucose before and after streptozotocin injection; **(B)** fasting insulin; **(C)** insulin resistance index (HOMA-IR); **(D)** blood glucose changes after oral 50% glucose solution; **(E)** HE staining of pancreatic tissue. Data were presented as mean ± SD (n = 8). Statistical differences were analyzed by two-tailed Student's t-test (**B** and **C**) or two - way ANOVA (**A** and **D**). **P < 0.01 and ****P <0.0001.

**Fig. (2):**
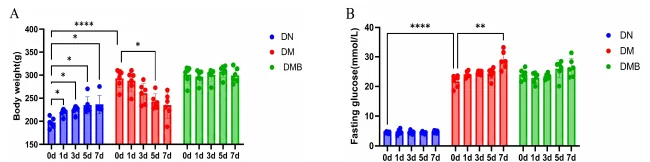
Effects of BP on body weight **(A)** and fasting glucose **(B)**. Data were presented as mean ± SD (n = 6). Statistical differences were analyzed by two-way ANOVA. *P < 0.05, **P < 0.01, and ****P < 0.0001.

**Fig. (3):**
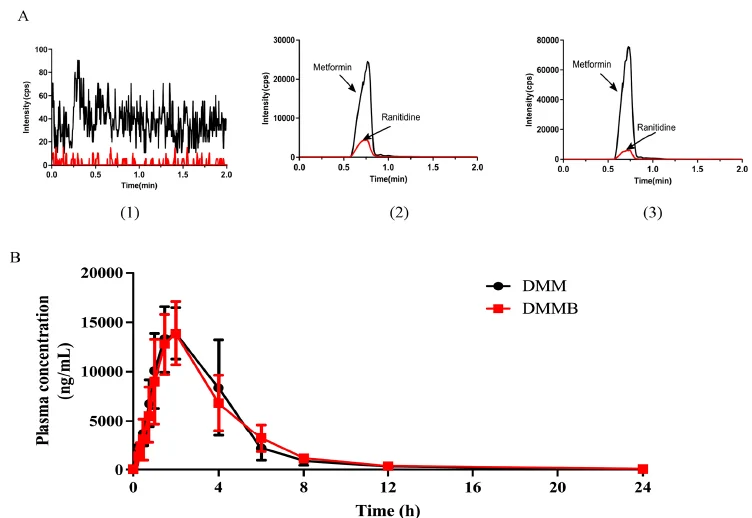
**(A)** Typical chromatograms of metformin and ranitidine in rat plasma samples. (1) blank plasma, (2) blank plasma mixed standard metformin and ranitidine solution and (3) plasma sample collected at 30 min after administration and ranitidine solution; **(B)** mean plasma concentration-time curves of metformin after administration between two group rats. Each data was presented as mean ± SD (n = 6).

**Fig. (4):**
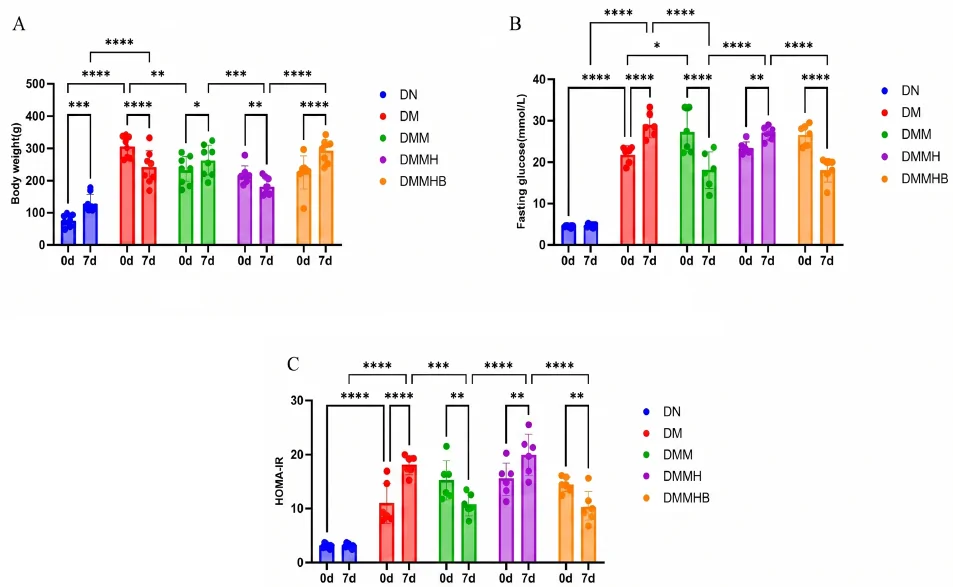
**(A)** Body weight (n=8), **(B)** fasting glucose (n=6), and **(C)** HOMA-IR (n=6). Data were presented as mean ± SD. Statistical differences were analyzed by two-way ANOVA. *P < 0.05, **P < 0.01, ***P < 0.001, and ****P < 0.0001.

**Fig. (5):**
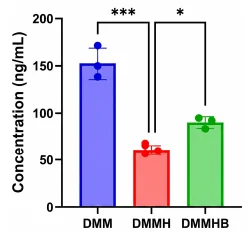
Metformin concentration in liver tissue. Data were presented as mean ± SD (n=3). Statistical differences were analyzed by one-way ANOVA. *P < 0.05 and ***P < 0.001.

**Fig. (6):**
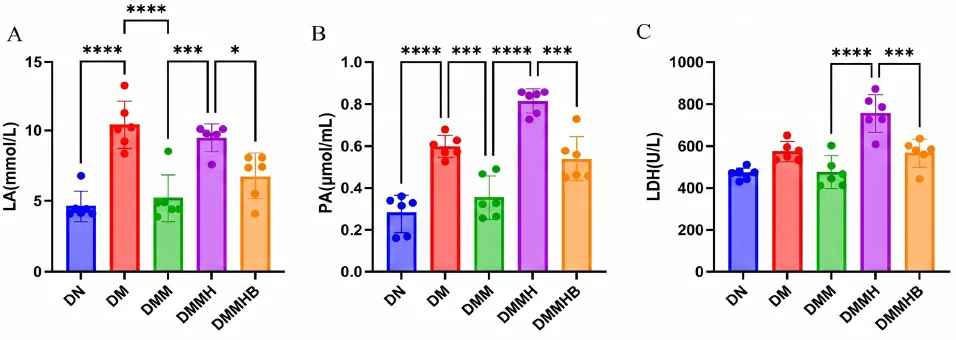
**(A)** Lactic acid (LA), **(B)** pyruvate **(**PA), and **(C)** lactate dehydrogenase (LDH) in serum. Data were presented as mean ± SD (n=6). Statistical differences were analyzed by one-way ANOVA. *P < 0.05, ***P < 0.001 and ****P < 0.0001.

**Fig. (7):**
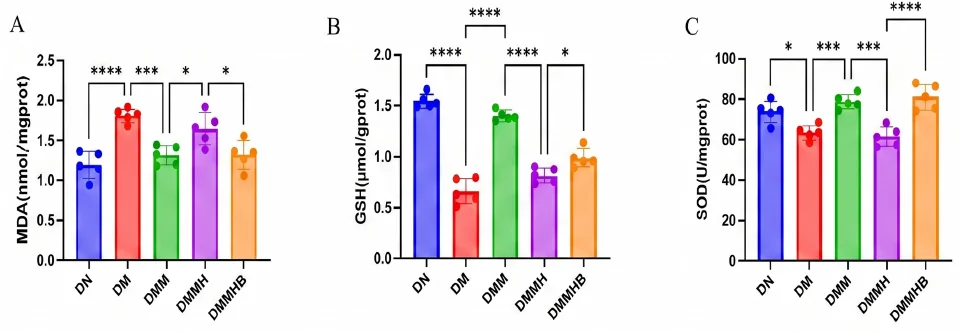
**(A**) Malondialdehyde (MDA), **(B)** reduced glutathione (GSH), and **(C)** superoxide dismutase (SOD) in liver tissues. Data were presented as mean ± SD (n=5). Statistical differences were analyzed by one-way ANOVA. *P < 0.05, ***P < 0.001, and ****P < 0.0001.

**Fig. (8):**
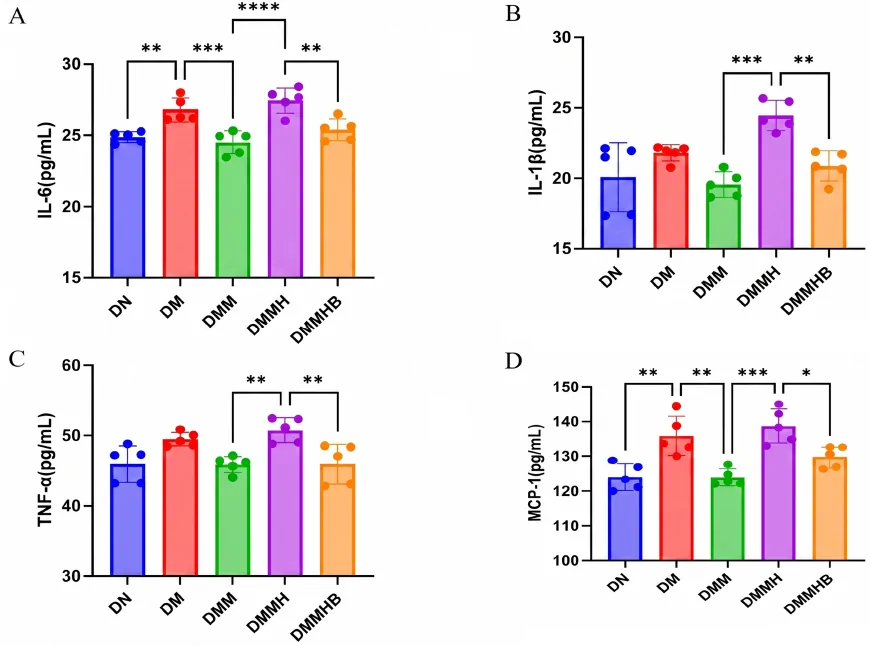
**(A)** Interleukin-6 (IL-6), **(B)** interleukin-1β (IL-1β), **(C)** tumor necrosis factor- α (TNF-α), and **(D)** monocyte chemoattractant protein-1 (MCP-1) in liver tissues. Data were presented as mean ± SD (n=5). Statistical differences were analyzed by one-way ANOVA. *P < 0.05, **P < 0.01, ***P < 0.001 and ****P < 0.0001.

**Table 1:** PK parameters of metformin in DMM and DMMB group.

Parameters	Units	DMM	DMMB
T_max_	h	1.83±0.24	1.75±0.38
C_max_	μg/mL	14.46±3.12	14.02±3.03
t_1/2_	h	3.77±0.95	3.93±1.17
V	L/kg	7.96±2.57	8.33±2.56
CL	L/h/kg	1.50±0.41	1.47±0.29
MRT_(0-t)_	h	3.53±0.36	3.80±0.31
MRT _(0-∞)_	h	3.67±0.31	4.03±0.27
AUC_(0-t)_	h*μg/mL	57.69±17.57	56.14±11.92
AUC _(0-∞)_	h*μg/mL	58.00±17.69	56.61±11.90

**Note:** Data were presented as mean ± SD (n = 6).

**Abbreviations:** T_max_, peak time; C_max_, peak concentration; t_1/2_, half-life; V, apparent volume of distribution; CL, clearance; MRT, mean residence time; AUC, area under the curve.

## Data Availability

All data generated or analysed during this study were included in this published article.

## LIST OF ABBREVIATIONS

BP: Betel Nut Polyphenols
CE: Collision Energies
DP: Declustering Potential
GSH: Reduced Glutathione
HOMA-IR: Homeostasis Model Assessment of Insulin Resistance (Insulin Resistance Index)
IL-6: Interleukin-6
IL-1β: Interleukin-1β
IS: Internal Standard
LA: Lactic Acid
LDH: Lactate Dehydrogenase
MDA: Malondialdehyde
MCP-1: Monocyte Chemoattractant Protein-1
PK: Pharmacokinetic
PD: Pharmacodynamic
PA: Pyruvate
SOD: Superoxide Dismutase
T2DM: Type 2 Diabetes Mellitus
TNF-α: Tumor Necrosis Factor-α
