# Supplementary material for: Enhancement of Metformin’s Efficacy by Betel Nut Polyphenols in Type 2 Diabetic Rats Exposed to High Altitude
Source: Endocr Metab Immune Disord Drug Targets. 2026 Jun 3;26:E18715303429512. doi: 10.2174/0118715303429512251203124106 (PMC13670197; doi:10.2174/0118715303429512251203124106)
Supplement: Supplementary file 1 [file EMIDDT-26-E18715303429512_SD1.pdf]

Supplementary Material

Enhancement of Metformin's Efficacy by Betel Nut Polyphenols in Type 2 Diabetic Rats Exposed to High Altitude

Xiaojing Zhang[1,#], Lin Luo[1,#], Wenbin Li[2], Hongfang Mu[2] and Rong Wang[1,*]

[1]Clinical Pharmacy of the 940th Hospital of Joint Logistic Support Force of Chinese People's Liberation Army, Lanzhou, China; [2]Pharmacy of the 940th Hospital of Joint Logistic Support Force of Chinese People's Liberation Army, Lanzhou, China

1. Supplementary Experimental Procedures

1.1 Linearity

The stock standard solution was diluted into standard curve samples of different concentrations of metformin (0.025~20 μg/mL) with rat blank plasma as the matrix by half dilution method. The metformin concentration was X, and the ratio of metformin peak area to IS peak area was Y, and then the standard curve equation of metformin in rat plasma was obtained by linear regression operation using the weighted least squares method.

1.2 Precision and accuracy

Quality control (QC) samples (low-0.025μg/mL, medium-1μg/mL and high-15 μg/mL) of metformin in rat plasma were analysed on the same day and on the three consecutive different days to measure the intra-day and inter-day precision and accuracy. Accuracy was expressed as the percentage of the measured value to the true value, and precision was expressed as the relative standard deviation (RSD) of the QC samples.

1.3 Extraction recovery and matrix effect

The following equations (1) and (2) were used to measure the extraction recovery and matrix effect of metformin:

$$Extraction\ recovery(\%) = \frac{peak\ area\ of\ analyte\ spiked\ blank\ plasma\ before\ extraction}{peak\ area\ of\ analyte\ spiked\ blank\ plasma\ after\ extraction} \times 100\% \quad (1)$$

$$\boldsymbol{Matrix\ effect}(\%) = \frac{\boldsymbol{peak\ area\ of\ analyte\ spiked\ blank\ plasma}}{\boldsymbol{peak\ area\ of\ analyte\ spiked\ aqueous\ solution}} \times \mathbf{100\%} \quad (2)$$

1.4 Stability

Short-term stability (25°C for 24 h), long-term stability (−80 °C for 10 days) and three freeze-thaw cycles stability of metformin in QC samples under different storage conditions were measured in this study. Stabilities were expressed as the concentrations after different operations to the concentration at time zero.

2. Supplementary Experimental Results

2.1 Linearity

When the metformin concentration was in the range of 0.025~20 μg/mL, the linear regression equation was Y=0.9744X+0.11 (R^2=0.9971). The limit of quantification of metformin was 25 ng/mL (S/N>10).

2.2 Precision and accuracy

The intra-day and inter-day precisions and accuracies of metformin were shown in Table S1. The intra-day and inter-day precisions were 2.1 %~4.4 % and 1.7 %~5.9 %, respectively, and the accuracies were 97.3 %~100.8 % and 98.2 % ~ 100.1 %, respectively. The assay for metformin was validated with an acceptable within 10 % (<15%) and met the methodological requirements.

2.3 Extraction recoveries and matrix effects

The recoveries and matrix effects of metformin were shown in Table S2, the extraction recoveries were 90.8 % ~ 96.1 % and the matrix effects were 90.1 % ~ 93.1%.

2.4 Stability

The results of the stability of metformin under different storage conditions were shown in Table S3. The long-term, three freeze-thaw cycles and short-term stabilities of metformin in rat plasma samples were between 92.3 % to 99.9 %. In addition, we also determined the stability of metformin in 85% acetonitrile solution after 24 h at 25°C and the results showed that the recovery of metformin was 97.3 % ~ 100.9 % (Table S3).

In summary, this method validation included good linearity, precision, accuracy, recovery and stability. These results indicated that metformin had good stability during sample preparation and analysis.

Table S1. Intra-day and inter-day precisions and accuracies for the analysis of metformin (n=6).

Concentration	Intra-day (%)		Inter-day (%)	
(μg/mL)	Precision (RSD)	Accuracy	Precision (RSD)	Accuracy
0.025	3.9	97.3	5.9	98.2
1	4.4	99.8	3.9	100.1
15	2.1	100.8	1.7	99.9

Table S2. Extraction recoveries and matrix effects of metformin.

Concentration	Extraction recovery (%)		Matrix effect (%)	
(μg/mL)	Average	RSD	Average	RSD
0.025	2.1	90.8	2.5	90.1
1	5.9	93.9	3.3	93.1
15	3.3	96.1	2.8	92.9

Table S3. Stabilities of metformin under different storage conditions.

Concentration	In rat plasma (%)			In 85% acetonitrile solution (%)
(μg/mL)	−80°C for 10 days	Freezing-thaw 3 cycles	25°C for 24 h	25°C for 24 h
0.025	93.5±6.0	92.3±4.4	96.7±3.1	97.3±6.3
1	97.2±4.5	96.8±3.3	99.9±0.8	98.5±0.8
15	99.3±1.1	97.3±2.7	98.7±0.5	100.9±1.9

Data were presented as mean ± SD (n = 3).
